# Tensile force impairs lip muscle regeneration under the regulation of interleukin‐10

**DOI:** 10.1002/jcsm.13584

**Published:** 2024-10-01

**Authors:** Xu Cheng, Jinfeng Dou, Jinggui Li, Yixuan Huang, Bing Shi, Jingtao Li

**Affiliations:** ^1^ State Key Laboratory of Oral Diseases, National Center for Stomatology, National Clinical Research Center for Oral Diseases West China Hospital of Stomatology, Sichuan University Chengdu China; ^2^ Department of Oral and Maxillofacial Surgery, State Key Laboratory of Oral Diseases, National Center for Stomatology, National Clinical Research Center for Oral Diseases West China Hospital of Stomatology, Sichuan University Chengdu China; ^3^ Department of Oral and Craniomaxillofacial Surgery Shanghai Ninth People's Hospital, Shanghai Jiao Tong University School of Medicine Shanghai China; ^4^ Shanghai Key Laboratory of Stomatology, National Center for Stomatology, National Clinical Research Center for Oral Diseases College of Stomatology, Shanghai Jiao Tong University Shanghai China

**Keywords:** inflammation mediators, mesenchymal stromal cells, orofacial cleft, satellite cells, skeletal muscle, surgical procedures, operative

## Abstract

**Background:**

Orbicularis oris muscle, the crucial muscle in speaking, facial expression and aesthetics, is considered the driving force for optimal lip repair. Impaired muscle regeneration remains the main culprit for unsatisfactory surgical outcomes. However, there is a lack of study on how different surgical manipulations affect lip muscle regeneration, limiting efforts to seek effective interventions.

**Methods:**

In this study, we established a rat lip surgery model where the orbicularis oris muscle was injured by manipulations including dissection, transection and stretch. The effect of each technique on muscle regeneration was examined by histological analysis of myogenesis and fibrogenesis. The impact of tensile force was further investigated by the in vitro application of mechanical strain on cultured myoblasts. Transcriptome profiling of muscle satellite cells from different surgical groups was performed to figure out the key factors mediating muscle fibrosis, followed by therapeutic intervention to improve muscle regeneration after lip surgeries.

**Results:**

Evaluation of lip muscle regeneration till 56 days after injury revealed that the stretch group resulted in the most severe muscle fibrosis (*n* = 6, fibrotic area 48.9% in the stretch group, *P* < 0.001, and 25.1% in the dissection group, *P* < 0.001). There was the lowest number of Pax7‐positive nuclei at Days 3 and 7 in the stretch group (*n* = 6, *P* < 0.001, *P* < 0.001), indicating impaired satellite cell expansion. Myogenesis was impaired in both the transection and stretch groups, as evidenced by the delayed peak of centrally nucleated myofibers and embryonic MyHC. Meanwhile, the stretch group had the highest percentage of Pdgfra^+^ fibro‐adipogenic progenitors infiltrated area at Days 3, 7 and 14 (*n* = 6, *P* = 0.003, *P* = 0.006, *P* = 0.037). Cultured rat lip muscle myoblasts exhibited impaired myotube formation and fusion capacity when exposed to a high magnitude (ε = 2688 μ strain) of mechanical strain (*n* = 3, *P* = 0.014, *P* = 0.023). RNA‐seq analysis of satellite cells isolated from different surgical groups demonstrated that interleukin‐10 was the key regulator in muscle fibrosis. Administration of recombinant human Wnt7a, which can inhibit the expression of interleukin‐10 in cultured satellite cells (*n* = 3, *P* = 0.041), exerted an ameliorating effect on orbicularis oris muscle fibrosis after stretching injury in surgical lip repair.

**Conclusions:**

Tensile force proved to be the most detrimental manoeuvre for post‐operative lip muscle regeneration, despite its critical role in correcting lip and nose deformities. Adjunctive biotherapies to regulate the interleukin‐10‐mediated inflammatory process could facilitate lip muscle regeneration under conditions of high surgical tensile force.

## Introduction

The orbicularis oris (OO) muscle serves as the sphincter encircling the oral fissure.^1^ It plays crucial roles in speaking, expression and maintaining facial aesthetics. In patients with congenital deformities, such as cleft lip and hemifacial microsomia, the OO muscle is usually disrupted, resulting in speech impairment and landmark distortions.^2^, ^3^ Because of its critical role in orofacial function and morphology, the OO muscle remains the key element in surgical lip repair.^4^, ^5^ Plastic surgeons regard the OO muscle reposition as the driving force for the repair of the other components.^6^, ^7^ However, the OO muscle frequently undergoes suboptimal regeneration after lip repair.^3^ Although a satisfactory surgical outcome could be achieved immediately after the surgery, as evidenced by a prominent vermilion tubercle and philtral ridge projection, a tight and notched lip can appear in long‐term observation.^4^, ^8^ These unsatisfactory results could be attributed to impaired muscle regeneration^9^ and the scar‐shortening effects: The balance of myogenesis and fibrogenesis during muscle regeneration was biased to the latter during the healing process. But the underlying mechanism remains elusive. This restricts further improvement in lip repair outcomes.

It is prerequisite to find a suitable model to study OO muscle regeneration. Current skeletal muscle regeneration models are inappropriate for two reasons. First, orofacial muscles are ontogenetically and phylogenetically distinct from limb and trunk muscles.^10^ The two groups of muscles exhibit marked differences in their developmental trajectory, myogenic regulatory factors, regenerative capacity and susceptibility to muscular diseases.^11^, ^12^ Consequently, limb and trunk muscle models, which comprise the vast majority of skeletal muscle regeneration models in the literature, are not pertinent to orofacial muscle studies. Moreover, heterogeneity exists among branchiomeric orofacial muscles,^13^, ^14^ making them a complex group to study. As a secondary branchial arch‐derived muscle,^13^ the OO muscle was never investigated before in terms of its regeneration capacity. Second, injury stimuli, including toxins, freezing, crushing and ischaemia re‐perfusion, were extensively investigated in the muscle studies.^15^ Nevertheless, the effects of surgical manoeuvres were seldom explored. In typical lip repair procedures, the OO muscle is exposed and located with multiple dissection techniques. Then the OO muscle was sutured with considerable force to correct the lip and nose deformities caused by aberrant muscle fibre insertion.^6^ It is unclear how these manipulations, like dissection, incision, transection and suture, will influence muscle regeneration.

In this study, a rat OO muscle injury model was established to recapitulate the authentic surgical manipulations in lip repair. The exclusive effect of each single manoeuvre was investigated with multi‐level biochemical analysis to figure out the one with the most detrimental influence. Further bioinformatic analysis and drug intervention studies revealed the relevant molecular changes in surgery‐induced OO muscle fibrosis. This work identified stretch as a critical negative regulator of post‐operative muscle regeneration, where interleukin‐10 (Il10) played a key regulatory role.

## Methods

### Animals

Adult male Sprague–Dawley (SD) rats (8 weeks, 280–300 g) were purchased from Chengdu Dashuo Biological Technology Company, China. All rats were housed in a humidity‐controlled (50 ± 5%) and temperature‐controlled (21 ± 2°C) facility with a 12‐h light/dark cycle. All experimental procedures on animals were approved by the Institutional Animal Care and Use Committee (IACUC, protocol number: WCHSIRB‐D‐2020‐114) at Sichuan University and have been performed in accordance with the ethical standards laid down in the 1964 Declaration of Helsinki and its later amendments. After 1‐week acclimation, only healthy rats were included in the following studies. The rats were randomly distributed into four groups: blank, dissection, transection and stretch groups. Detailed information on the animal distributions is listed in *Table* *S1*.

### Surgical manipulation of rat orbicularis oris muscle

**Figure 1:**
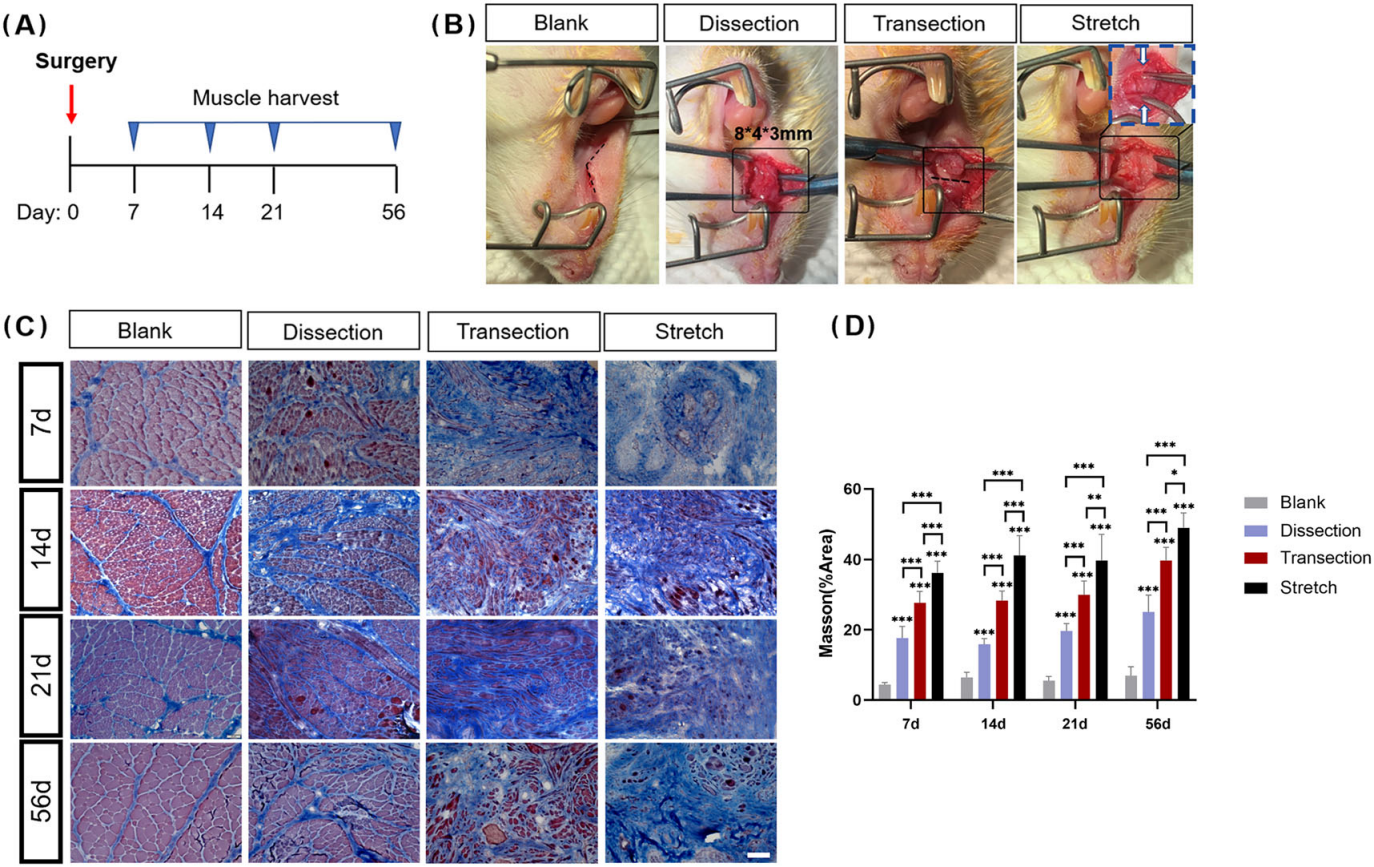
The orbicularis oris (OO) muscle exhibited fibrotic alterations after surgical manipulation. (A) Schematic overview of the surgical injury and muscle harvest timeline. (B) Illustration of the dissection, transection and stretch procedures in the OO muscle. The black boxes indicated the surgical field. The black dotted line indicated the incision of the muscle bundle. The space between the two white arrows in the blue dotted line indicated the OO muscle defect after muscle bundle removal. The white arrows pointed to the residual ends of the OO muscle, where sutures were to be made. (C) Masson trichrome staining of the OO muscle at the centre of the injury harvested at 7, 14, 21 and 56 dpi. (D) Quantification of the fibrosis area. Dpi, days post‐injury. Scale bar, 50 μm. *P < 0.05; **P < 0.01; ***P < 0.001.

Sedate the rats with isoflurane‐soaked cotton balls and apply an intraperitoneal injection of Zoletil at a dose of 50 mg/kg. Protect their eyes with vet ointment. Fix the rats in a supine position on a surgical table. Access to the OO muscle was done as previously described.^16^ Briefly, a mouth gag was used to expose the lateral lip (*Figure* *1* *A*). The incision was made along the junction between the vermilion and mucosa. The OO muscle was separated from the skin and mucosa with forceps and scissors, creating an 8 * 4 * 3‐mm muscle bundle (*Figure* *1* *A*). In the dissection group, sutures were applied right after this step (*Figure* *1* *A*). In the transection group, an incision in the OO muscle bundle was made, followed by end‐to‐end suturing and skin closure (*Figure* *1* *A*). In the stretch group, a segment of a 4 * 4 * 3‐mm muscle bundle was cut and removed. And the remaining broken muscle ends were sutured, followed by skin closure (*Figure* *1* *A*). At different timepoints post‐injury, the OO muscle was harvested for subsequent histological analysis. The dissection manual was described in detail in a previous paper.^16^ Briefly, the OO muscle could be identified by the attachment of the perioral muscles to the premaxilla and the buccal fat pad. Isolate the OO muscle bundle by disrupting the myomucosal junction with a sharp dissection. The suture was designated as the centre of the injury.

### Tissue preparation, histology and immunohistochemistry

Harvested rat OO muscle was fresh and frozen, and cryosections were made according to Lawlor's method.^17^ Muscle cryosections were made at 10 μm and fixed in ice‐cold acetone for 20 min. The sections were stained with Masson's Trichrome or Picro Sirius Red staining kit (Solarbio). For immunofluorescent staining, sections were treated with a blocking solution and incubated overnight at 4°C with primary antibodies. The next day, the sections were incubated for 1 h at room temperature with secondary antibodies. The nuclei were stained with 4′,6‐diamidino‐2‐phenylindole (DAPI). Images were captured with an Olympus BX63 fluorescence microscope (Olympus Corporation, Tokyo, Japan). The primary antibodies used in this study were as follows: laminin (L9393, Sigma‐Aldrich), Pdgfra (AF062, R&D Systems), Pax7 (Pax7, DSHB) and emb‐MyHC (BF‐G6, DSHB). The secondary antibodies used in this experiment were Alexa Fluor 488 donkey anti‐goat (ab150129, Abcam), Alexa Fluor 568 donkey anti‐mouse (A10037, Invitrogen), donkey anti‐rabbit FITC (ZF‐0311, ZSGB‐BIO) and donkey anti‐goat TRITC (ZF‐0316, ZSGB‐BIO).

### Cell isolation and culture

The OO muscle from adult male SD rats (8 weeks, 280–300 g) was dissected as described before.^16^ Muscle satellite cells (MuSCs) were isolated and cultured in vitro according to Paola's method with some modifications.^18^ The isolated OO muscle was minced into 1–2 mm^3^ of fragments after nerves, vessels and fascia were removed. The muscle slurry was digested in 0.1% Pronase at 37°C for 1 h. The MuSCs were released by trituration and centrifugation. The cell pellet was resuspended in growth medium: high‐glucose Dulbecco's modified Eagle's medium (DMEM) supplemented with 20% foetal bovine serum (FBS), 10% horse serum (HS), 100 U/mL penicillin and 100 μg/mL streptomycin. The cells were seeded in a Matrigel‐coated six‐well plate at a density of 2 × 10^5^ per well. The culture medium was refreshed every 2–3 days. The cells were passaged at 70–80% confluency. P2–P4 cells were used for subsequent experiments. Fibro‐adipogenic progenitors (FAPs) were isolated using the preplating method. Briefly, two‐step digestion was performed using collagenase II and dispase II. The cell suspension was plated in a T75 flask. After 3 h, the cells not attached were discarded. The attached cells were collected for downstream analysis.

### Recombinant human Wnt7a administration

For in vivo delivery, recombinant human Wnt7a (rh‐Wnt7a) was injected directly into the rat OO muscles with a Hamilton micro‐injection syringe. Rats were randomly distributed into three groups: (1) a control group without OO muscle manipulation; (2) a stretch group created as previously described; and (3) a Wnt7a group where the stretch‐injured OO muscles were injected with rh‐Wnt7a immediately and at Day 2 post‐injury. Rh‐Wnt7a was injected at 25 μL with a concentration of 100 μg/mL each time. For in vitro administration, rh‐Wnt7a was added to the MuSC culture medium at a concentration of 100 ng/mL.

### Mechanical strain application

A four‐point bending device (SXG4201; Chengdu Miracle Chemicals Co., Ltd., Chengdu, China) was used to apply strain on the cultured MuSCs. As previously described, the device was composed of a mechanical power system, a strain‐loading dish and a host computer. The power system could produce mechanical stretch on the dish, thus deforming the cells seeded here. Once the parameter of the dish was specified, the cell strain could be adjusted and controlled precisely by the host computer: ε = td/a(L − 1.33a), where ε is the cell strain; t is the thickness of the loading plate; d is the displacement; L is the distance between two outer pressure points; and a is the distance between inner and outer pressure points.

For cell proliferation analysis, MuSCs were seeded at a density of 2 × 10^5^ cells/mL in a 1.5‐mL groove and put in the incubator for 12 h in growth medium for optimal cell attachment. Then the culture dish was put on top of the stain‐loading dish and filled with 80 mL of growth medium. The cells were divided into four groups with different mechanical strains. The selected strain parameters are listed in *Table* *S2*. The duration of the strain application was 120 min. Cells were cultured for another 22 h before fixation and subsequent immunocytochemistry analysis.

For cell differentiation analysis, MuSCs were seeded at a density of 4.5 × 10^5^ cells/mL and put in the incubator in growth medium for 6 h. Then the culture medium was refreshed with differentiation medium (5% HS, 100 U/mL penicillin and 100 μg/mL streptomycin) and cultured for 12 h. Then the culture dish was put on top of the stain‐loading dish and filled with 80 mL of differentiation medium. The cells were divided into four groups with different mechanical strains. The selected strain parameters are listed in *Table* *S2*. The duration of the strain application was 120 min/day for 3 days. In the remaining 22 h of each day, cells were put in the incubator under normal culture conditions.

### Immunocytochemistry

At the end of mechanical strain loading, the cells were fixed in 4% paraformaldehyde (PFA) and permeabilized with 0.5% Triton‐X100. The cells were blocked with 5% bovine serum albumin and 5% donkey serum for 1 h at room temperature and incubated overnight at 4°C with MyHC (ab50967, Abcam) or Ki67 (ab1667, Abcam). The next day, the cells were incubated for 1 h at room temperature with Alexa Fluor 568 donkey anti‐mouse (A10037, Invitrogen) and donkey anti‐rabbit FITC (ZF‐0311, ZSGB‐BIO), respectively. Then the nuclei were stained with DAPI. Images were captured with an Olympus BX63 fluorescence microscope (Olympus Corporation).

### Flow cytometry and RNA‐seq

The OO muscles from blank, dissection, transection and stretch groups were harvested 4 days post‐injury. In each group, three rats with both sides of the OO muscle were harvested to create one sample. The isolated muscles were cut into small fragments on ice for following flow cytometry analysis. MuSCs were sorted by flow cytometry according to Rando's method, with some modifications.^19^ Briefly, minced muscle was transferred to a conical tube and underwent two rounds of digestion: first in 700–800 U/mL collagenase II muscle dissociation buffer for 1 h and then in 1000 U/mL collagenase type II and 11 U/mL dispase II for 30 min. After digestion, the muscle pellet was triturated using a 20‐gauge needle, centrifuged at 500 g for 5 min and passed through a 40‐mm cell strainer twice to obtain a single‐cell suspension. Cells were placed in labelled 2‐mL tubes and stained with fluorescent conjugated antibodies on a rotating mixer for 40 min at 4°C, resuspended in wash medium and filtered through a round‐bottom polystyrene test tube with a cell strainer snap cap. Cells were then sorted on a BD FACS Aria II flow cytometer. MuSCs were identified as VCAM1^+^CD31^−^CD45^−^Ter119^−^Sca1^−^ cells. The purity of the MuSCs was determined by Pax7 immunofluorescent staining. Total RNA of the sorted MuSCs was extracted by RNA‐easy™ Isolation Reagent (Vazyme, Nanjing, China). After quality control, raw reads were filtered by removing low quality, junction contamination and high unknown base N content to obtain clean reads. The clean reads were aligned to the reference genome for new transcript prediction, SNP and INDEL and differential splicing gene detection. After getting a complete reference sequence, the gene expression level was calculated. Finally, the differentially expressed genes among different samples were detected and subjected to clustering analysis and functional enrichment analysis.

### Quantitative real‐time PCR

RNA was extracted from OO muscle or cultured MuSCs using the RNeasy Mini Kit (QIAGEN). First‐strand cDNA was synthesized using Rayscript cDNA Synthesis Kit (GENEray, GK8030, Shanghai, China). Quantitative PCR was performed using the SYBR Green I master kit (Roche) on a LightCycler 480. The comparative cycle threshold (CT) was used to analyse the data by generating relative values of the amount of target cDNA as described. The expression level was normalized to GAPDH. All experiments were done in triplicate. The primers used in this experiment were as follows: MyoG forward: GCA GGC TCA AGA AAG TGA ATG A; MyoG reverse: TAG GCG CTC AAT GTA CTG GAT; Myod1 forward: TCC GCT ACA TCG AAG GTC TG; Myod1 reverse: GTC CAG GTG CGT AGA AGG C; Myf6 forward: GCC AAG TGT TTC GGA TCA TTC; Myf6 reverse: GGA GTT TGC GTT CCT CTG AG; Myf5 forward: CAC CAC CAA CCC TAA CCA GAG; Myf5 reverse: AGG CTG TAA TAG TTC TCC ACC TG; Tnf forward: CTT CTG TCT ACT GAA CTT CGG G; Tnf reverse: CTA CGG GCT TGT CAC TCG; Tgfb1 forward: CTT CAA TAC GTC AGA CAT TCG GG; Tgfb1 reverse: CTT CAA TAC GTC AGA CAT TCG GG; Pdgfra forward: GTT GCC TTA CGA CTC CAG ATG; Pdgfra reverse: TCA CAG CCA CCT TCA TTA CAG; Col3a1 forward: GAA GTC TCT GAA GCT GAT GGG; Col3a1 reverse: GGC CTT GCG TGT TTG ATA TTC; Col1a1 forward: AGC CGC AAA GAG TCT ACA TG; Col1a1 reverse: CTT AGG CCA TTG TGT ATG CAG; Acta2 forward: GGA CGT ACA ACT GGT ATT GTG C; Acta2 reverse: TCG GCA GTA GTC ACG AAG GA; Il1b forward: GAC AAG CAA CGA CAA AAT CCC; Il1b reverse: TGG GTA TTG TTT GGG ATC CAC; Il6 forward: AGA GTT GTG CAA TGG CAA TTC; Il6 reverse: AGA CCA GAG CAG ATT TTC AAT AGG; Myh1 forward: CGG AGT CAG GTG AAT ACT CAC G; Myh1 reverse: GAG CAT GAG CTA AGG CAC TCT; Il10 forward: TAA GGG TTA CCT TGG GTT GCC AAG CC; Il10 reverse: AGG GGA GAA ATC GAT GAC AGC GCC; Tgfb1 forward: CTG CTG ACC CCC ACT GAT AC; Tgfb1 reverse: AGC CCT GTA TTC CGT CTC CT; GAPDH forward: GAC ATG CCG CCT GGA GAA AC; GAPDH reverse: AGC CCA GGA TGC CCT TTA GT.

### Enzyme‐linked immunosorbent assay

IL‐10 protein concentration was assessed in the OO muscle tissue using an enzyme‐linked immunosorbent assay (ELISA) (Jiangsu Meimian, MM‐0195R1). Muscle tissue from blank, dissection, transection and stretch groups was homogenized in 0.01 mol/L phosphate‐buffered saline (PBS) (w/v 1:10) on ice with a homogenizer. The homogenate was centrifuged at 5000 rpm for 15 min at 4°C, and the supernatant was collected for protein concentration analysis according to the manufacturer's instructions. The same ELISA kit was used for measuring IL‐10 concentrations from MuSCs and FAPs.

### Statistical analysis

The sample size was calculated by power analysis.^20^ All rats were randomized according to body weight before interventions. Statistical analyses were performed using SPSS 19.0 or GraphPad Prism. A comparison of distributions was performed by a Kolmogorov–Smirnov test. For comparison of the four surgically manipulated groups and the mechanical strain groups, a one‐way analysis of variance (ANOVA) was used, followed by Bonferroni multiple‐comparison testing. Statistical significance for binary comparisons was assessed by a Mann–Whitney test. All exploratory and signalling experiments were analysed using two‐tailed tests. *P* < 0.05 was considered statistically significant. All data are expressed as the mean ± SEM.

## Results

### Surgical manipulations induced orbicularis oris muscle fibrosis

Surgical manipulations, including muscle dissection, transection and stretch, were performed on rat OO muscle. Muscle histology was assessed by Masson trichrome staining. On Day 7 after surgical injury, the OO muscle tissue exhibited obvious fibrosis in the dissection, transection and stretch groups in comparison to the control group (*Figure* *1* *C,D*). Particularly, fibrotic area remained highest (36%; *Figure* *1* *C,D*) in the stretch group and lowest in the dissection group (18%; *Figure* *1* *C,D*). A similar trend among different surgical groups was observed at longer timepoints, where the percentage of fibrotic area was always the largest in the stretch group (*Figure* *1* *D*). Temporal dynamics of the fibrotic area revealed that after surgical injury, the OO muscle fibrosis persisted and even increased with time, with 48.9% of the fibrotic area in the stretch group and 25.1% in the dissection group (*Figure* *1* *C,D*).

### Distinct temporal dynamics of myogenesis and fibrogenesis in response to different surgical manoeuvres

**Figure 2:**
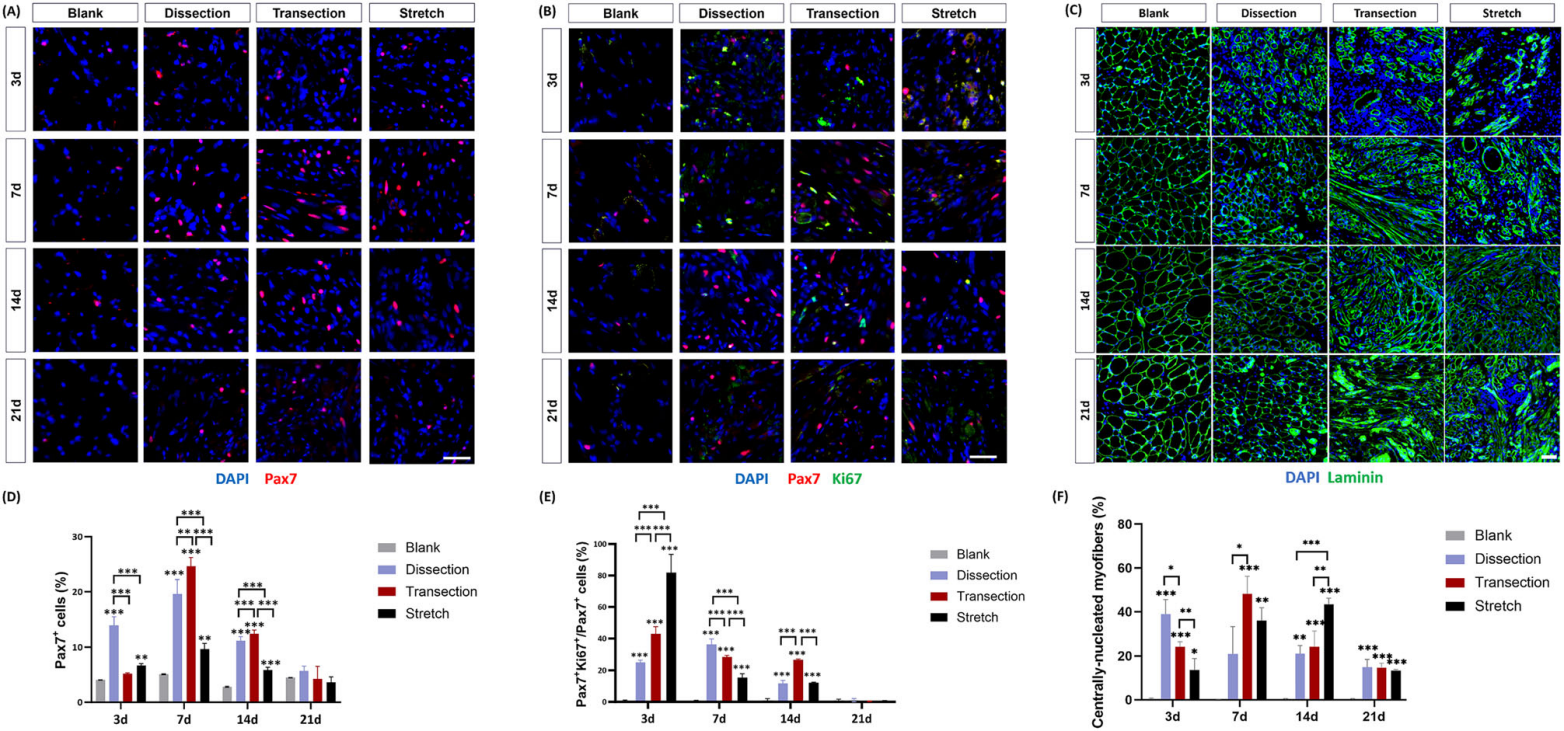
Comparison of muscle satellite cell (MuSC)‐mediated muscle regeneration in different surgical groups. (A) Immunofluorescent staining of 4′,6‐diamidino‐2‐phenylindole (DAPI) (blue) and Pax7 (red) in different groups. (B) Immunofluorescent staining of DAPI (blue), Pax7 (red) and Ki67 (green) in different groups. (C) Immunofluorescent staining of DAPI (blue) and laminin (blue) in different groups. (D–F) Quantification of Pax7^+^ satellite cells, Pax7^+^Ki67^+^ proliferative satellite cells and centrally nucleated myofibers. Scale bars in (A) and (B), 20 μm; scale bar in (C), 50 μm. *P < 0.05; **P < 0.01; ***P < 0.001.

**Figure 3:**
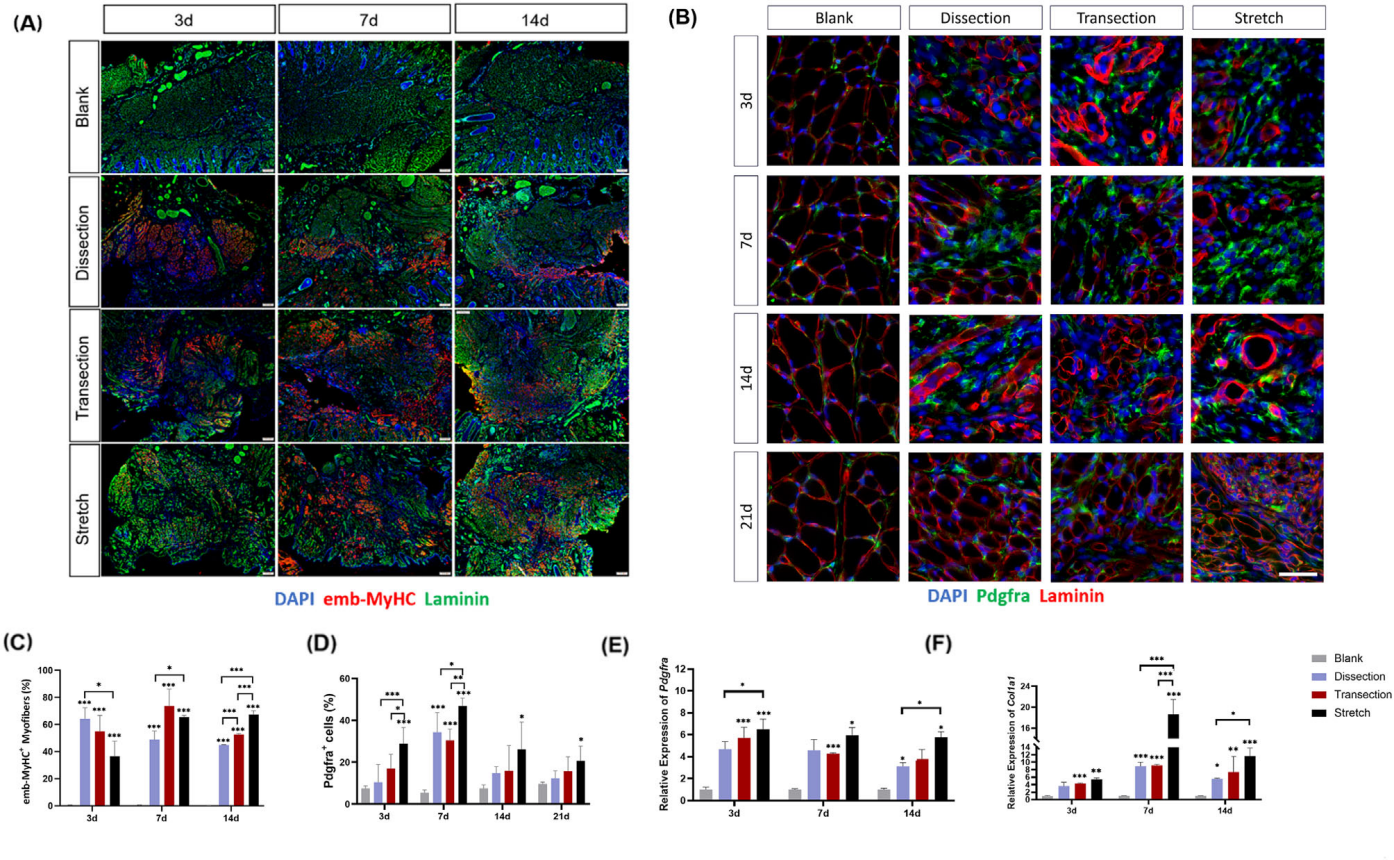
Temporal dynamics of myogenesis and fibrogenesis in different surgical groups. (A) Immunofluorescent staining of laminin (green), 4′,6‐diamidino‐2‐phenylindole (DAPI) (blue) and embryonic MyHC (emb‐MyHC) (red) in blank, dissection, transection and stretch groups. Scale bar, 200 μm. (B) Immunofluorescent staining of Pdgfra (green), DAPI (blue) and laminin (red) in blank, dissection, transection and stretch groups. Scale bar, 20 μm. (C, D) Quantification of emb‐MyHC and Pdgfra in immunofluorescence pictures. (E, F) Quantification of relative expression levels of Pdgfra and Col1a1 in blank, dissection, transection and stretch groups. *P < 0.05; **P < 0.01; ***P < 0.001.

To interrogate the mechanism underlying the tilted OO muscle regeneration towards fibrosis, we carried out a panel of immunofluorescent analyses to evaluate the myogenic and fibrogenic activities, both crucial in skeletal muscle regeneration. The proportion of Pax7‐positive MuSCs peaked at Day 7 post‐injury in all three groups and returned to the uninjured level at Day 21 (*Figure* *2* *A,D*). At Days 7 and 14, the transection group had the largest MuSC proportion, while the dissection group for Day 3. In contrast, the MuSC expansion was inhibited in the stretch group, as evidenced by a smaller proportion of Pax7^+^ nuclei at Days 3, 7 and 14 (*Figure* *2* *A,D*). The proportion of Pax7^+^Ki67^+^ proliferative MuSCs in the stretch group was the highest at Day 3, while at Day 7, the highest proportion appeared in the dissection group (*Figure* *2* *B,E*). Meanwhile, the quantification of centrally nucleated myofibers (CNMs), a hallmark of MuSC‐mediated muscle regeneration, demonstrated different temporal dynamics. The proportion of CNMs peaked at Day 3 in the dissection group, at Day 7 in the transection group and at Day 14 in the stretch group (*Figure* *2* *C,F*). The OO muscle myogenic activity was further evaluated by embryonic MyHC (emb‐MyHC) staining. The proportion of emb‐MyHC^+^ myofibers peaked at Day 3 in the dissection group, at Day 7 in the transection group and at Day 14 in the stretch group (*Figure* *3* *A,C*). On the other hand, the fibrogenic process was assessed by Pdgfra, the surface marker for FAPs. The percentage of Pdgfra^+^ nuclei peaked at Day 7 and returned to the control level at Day 21 in all three groups, except for the stretch group, where the high FAP number persisted (*Figure* *3* *B,D*). At each timepoint, the stretch group had the highest percentage of Pdgfra^+^ nuclei. This was further verified by the increased expression levels of *Pdgfra* and *Col1a1* (*Figure* *3* *E,F*). In addition, the relative expression levels of *Tgfb1* and markers for inflammation (*Tnfa*, *Il6* and *Il1b*) were significantly higher in the surgery groups in a stepwise way, with the stretch group having the highest (*Figure* *S1*).

Taken together, all three surgical manoeuvres activated myogenic and fibrogenic activities. In comparison to the dissection group, myogenesis was impaired in both the transection and stretch groups, as evidenced by the delayed peak of CNMs and emb‐MyHC. Fibrogenesis and inflammatory processes persisted in all three surgical groups, among which the stretch group had the highest level of fibrosis and inflammation.

### High mechanical strain inhibited myotube formation in vitro

**Figure 4:**
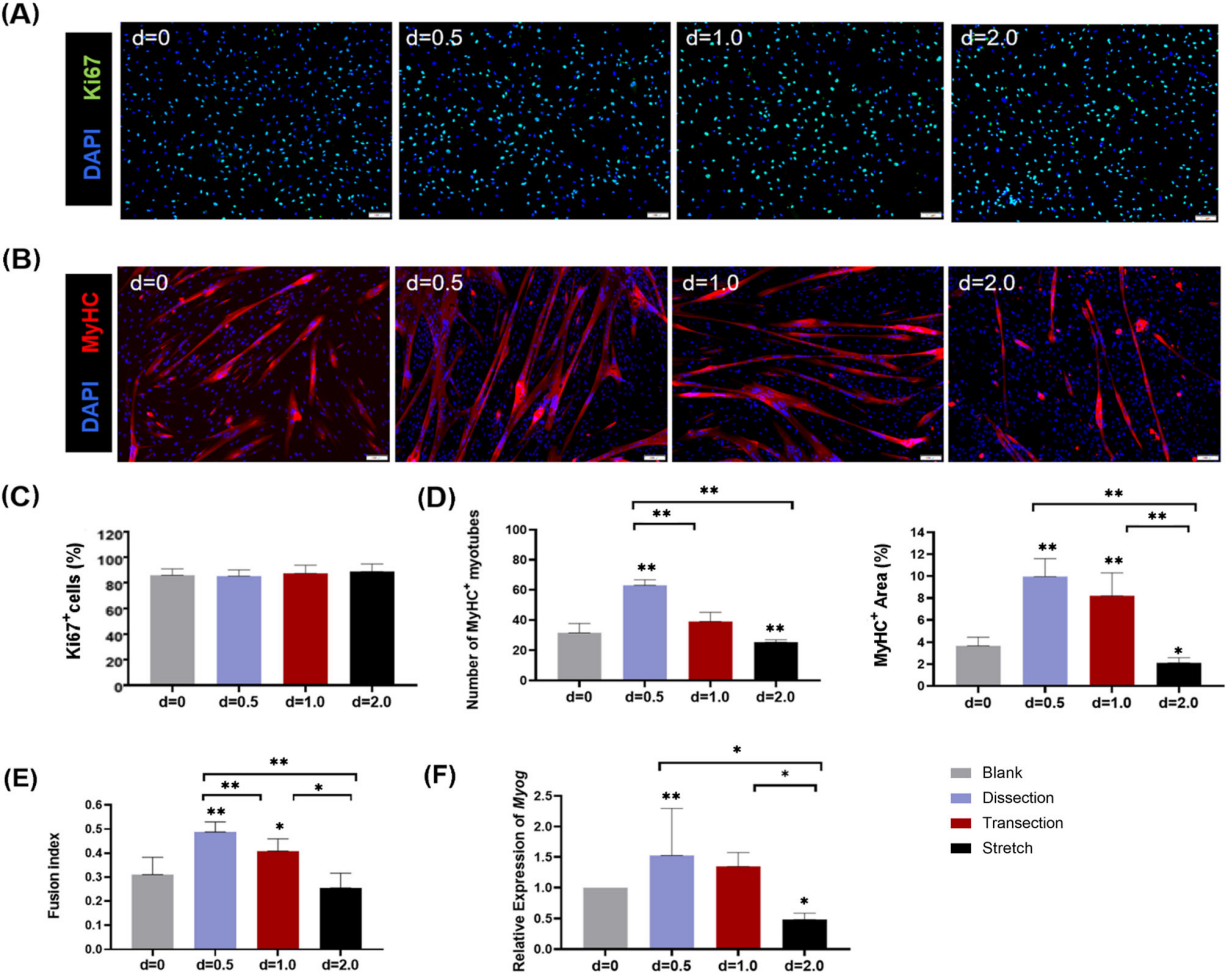
High mechanical strain impaired myogenesis in vitro. (A) Immunofluorescent staining of Ki67 (green) and DAPI (blue) in different groups with low, middle and high mechanical strain. (B) Immunofluorescent staining of MyHC (red) and DAPI (blue). (C) Quantification of Ki67 positive nuclei. (D) Quantification of MyHC positive myotubes and MyHC positive area. (E) Quantification of fusion index. (F) Quantification of relative expression level of MyoG. Scale bar, 50 µm.

Histological analysis revealed that stretch impaired OO muscle regeneration to the largest extent. Further in vitro studies were then carried out to characterize the role of stretch on the cellular behaviour of cultured myoblasts. Three different magnitudes of mechanical strains were applied to the OO muscle myoblast. Under different stretch stimuli, the myoblast exhibited no significant changes in cell proliferation (*Figure* *4* *A,C*) but demonstrated different alterations in myogenic differentiation and fusion (*Figure* *4* *B,D–E*). The low‐intensity strain (d = 0.5) significantly increased the number and size of OO muscle myotubes, as well as the fusion index (*Figure* *4* *B,D–* *E*). The middle‐intensity strain (d = 1.0) significantly increased the size and fusion index of the myotubes (*Figure* *4* *B,D–* *E*). The high‐intensity strain (d = 2.0), however, had an opposite effect on the myoblast: The size and number of myotubes were reduced, while myonuclei fusion was not affected (*Figure* *4* *B,D–* *E*). Transcriptional analysis of myogenic differentiation also demonstrated that the expression level of *Myog* was elevated in the low‐intensity strain group but decreased in the high‐intensity strain group (*Figure* *4* *F*).

### *Il10* plays a key role in orbicularis oris muscle satellite cell transcriptional changes after surgery

To uncover the molecular features of the altered myoblast behaviour after different surgical manoeuvres, we profiled the transcriptome of FACS‐sorted OO MuSCs at 4 dpi of blank, dissection, transection and stretch groups. The Vcam1^+^Sca1^−^ cells were designated as MuSCs. Immunofluorescent staining revealed that more than 96% of the sorted cells were Pax7 positive (*Figure* *S2*).

**Figure 5:**
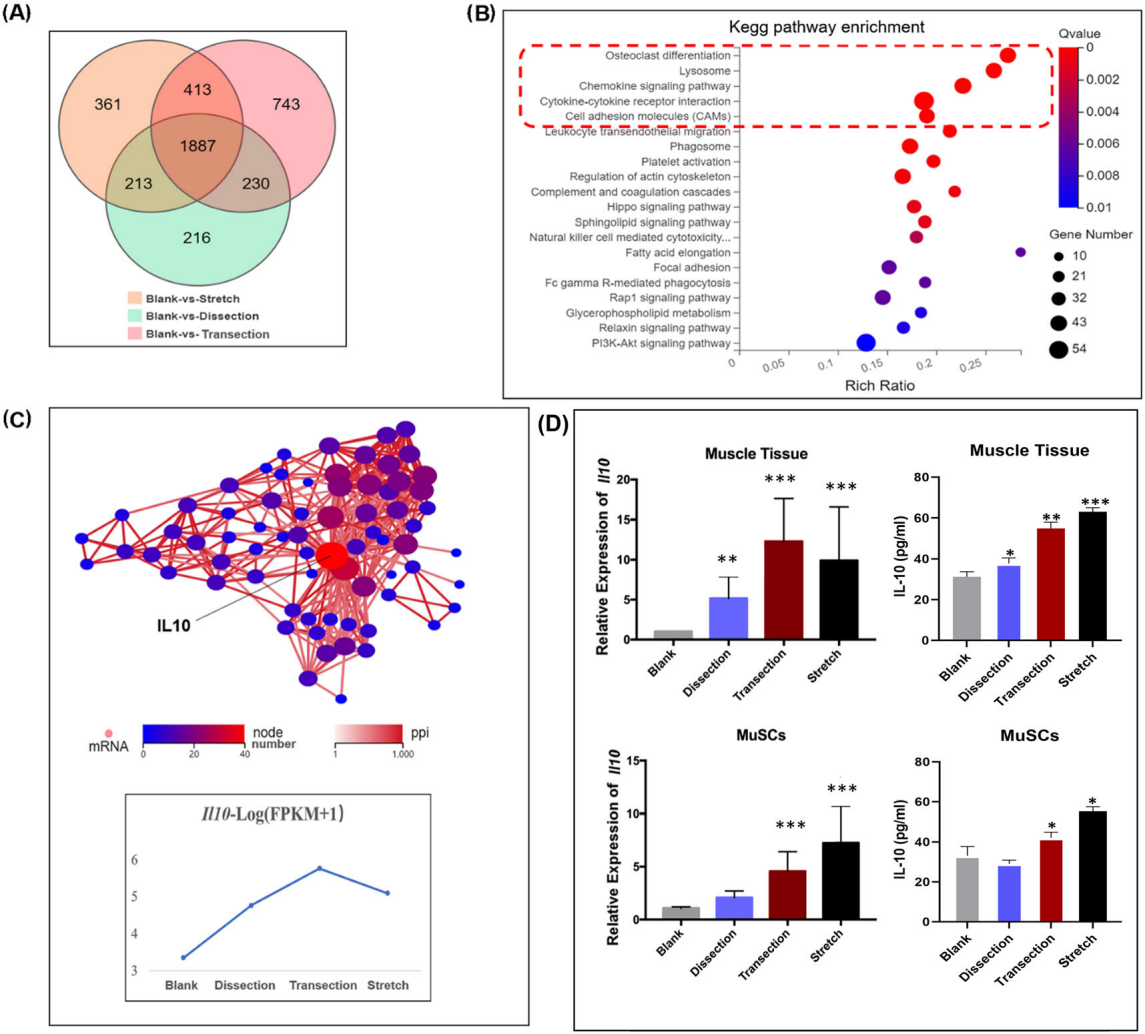
Muscle satellite cell (MuSC) transcriptome profiling reveals IL10 as a key regulator. (A) Venn diagram of genes differentially regulated between the injured groups (dissection, transection and stretch groups) and the blank group. (B) Analysis of the Kyoto Encyclopedia of Genes and Genomes (KEGG) pathway enrichment in the 1877 genes commonly expressed in the injured groups. (C) Protein–protein interaction of the top five pathways in KEGG enrichment and the expression level of IL10 in different groups. (D) Quantitative real‐time PCR of *Il10* and analysis of protein levels of IL‐10 in orbicularis oris (OO) muscle tissue and MuSCs from different groups.

RNA‐seq analysis identified a total of 4063 genes differentially expressed in the three injury groups (|log2FC| ≥ 1 and Q value ≤ 0.05). A total of 1887 genes were commonly expressed by all three groups (*Figure* *5* *A*). The Kyoto Encyclopedia of Genes and Genomes (KEGG) pathway enrichment revealed that they had strong signatures of ‘osteoclast differentiation’, ‘lysosome’, ‘chemokine signaling pathway’, ‘cytokine‐cytokine receptor interaction’ and ‘cell adhesion molecules’ (*Figure* *5* *B*). The genes that belong to these top five categories were explored with Gene Ontology (GO) enrichment and protein–protein interaction (PPI) analysis. GO terms enriched in immune responses ranked at the top. Interestingly, PPI analysis identified *Il10* as the hub, with an increased expression in all three injury groups (*Figure* *5* *C*). Quantitative real‐time PCR analysis of *Il10* and ELISA analysis of IL‐10 were then performed in sorted MuSCs, FAPs and isolated OO muscle tissue at 4 dpi. The results revealed that the relative expression levels of *Il10* and IL‐10 protein levels were significantly increased in groups with surgical manipulations (*Figures* *5* *D* and *S3*).

### Wnt7a ameliorates stretch‐induced orbicularis oris muscle fibrosis

**Figure 6:**
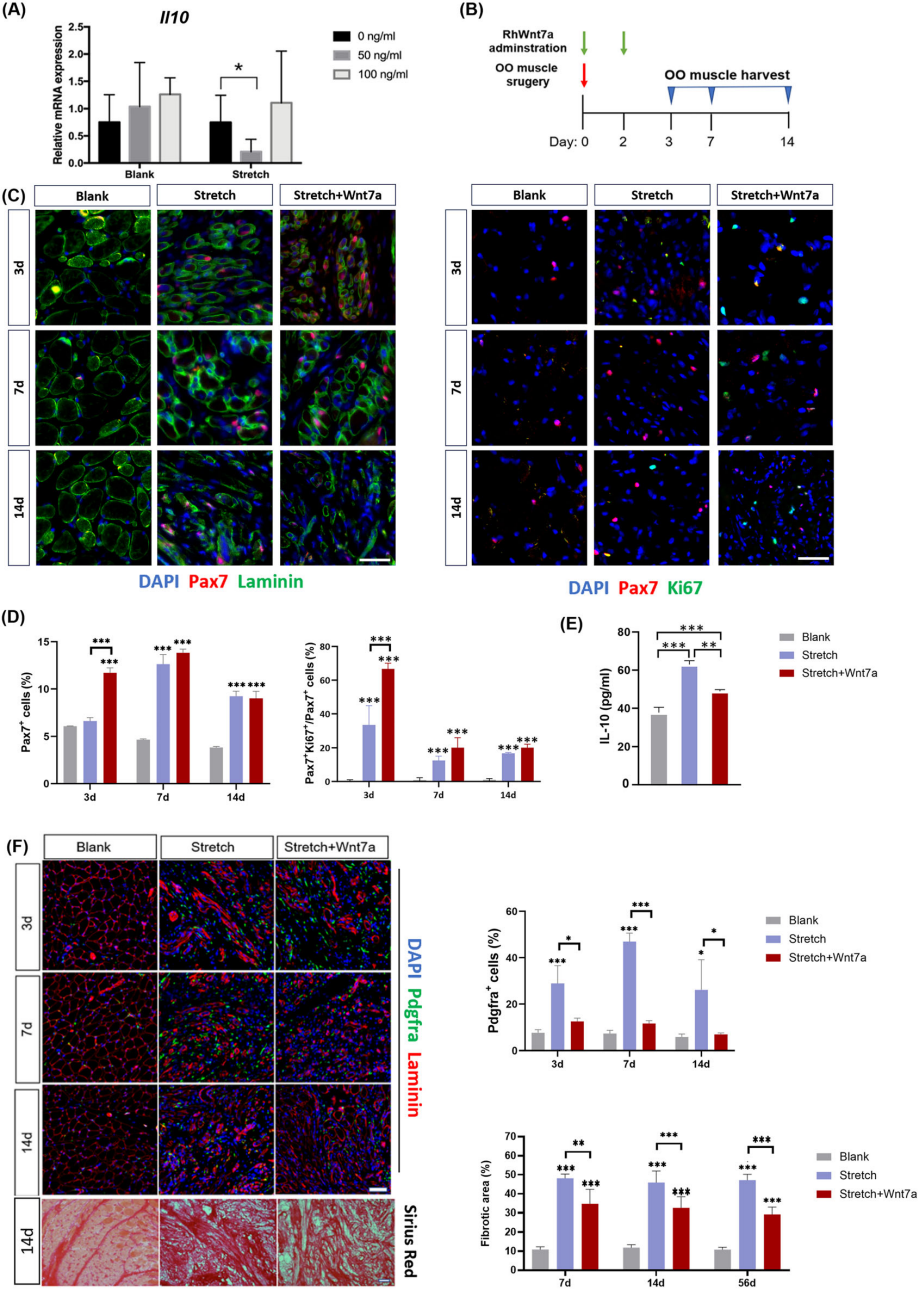
Injection of rh‐Wnt7a ameliorates stretch‐induced OO muscle fibrosis. (A) Comparison of Il10 expression in blank or stretch‐induced OO muscle myoblast with or without rh‐Wnt7a administration. (B) Stretch injury was performed in rat OO muscle, followed by intramuscular rh‐Wnt7a injection twice, immediately and 2 days after injury. OO muscles were harvested at day 3, day 7 and day 14 post‐injury. (C) Immunofluorescent staining of DAPI (blue), Pax7 (red) and laminin (green)/Ki67(green) in different groups. (D) Quantification of Pax7^+^ satellite cells and Pax7^+^Ki67^+^ proliferative satellite cells. (E) Quantitative analysis of IL‐10 protein in different groups. (F) Immunofluorescent staining of DAPI (blue), laminin (red) and Pdgfra (green) and histological staining of Picro Sirius Red in different groups in the left panel. Quantification of Pdgfra^+^ FAP cells and of fibrotic area in the right panel. Scale bars in (C), 20 µm; scale bars in (F), 50 µm.*P < 0.05; **P < 0.01; ***P < 0.001.

Because the Wnt pathway plays a critical role in regulating immune response^21^ and Wnt7a has been proven efficient in promoting orofacial muscle regeneration,^22^ we intend to test the effects of Wnt7a in rat OO muscle regeneration after surgical stretch. The regulatory role of Wnt7a was first investigated in vitro. In the myoblasts from blank muscles, rh‐Wnt7a did not significantly change the relative expression level of *Il10*, while in the MuSCs from stretched muscles, Wnt7a inhibited *Il10* expression, but only in the low concentration group (*Figure* *6* *A*). In vivo rh‐Wnt7a delivery revealed that Wnt7a significantly reduced the IL‐10 protein concentration, which was increased after stretch manipulation (*Figure* *6* *B*,*E*). In addition, Wnt7a affected both OO muscle myogenesis and fibrogenesis. The proportion of Pax7^+^ MuSCs increased at Day 3 after Wnt7a administration (*Figure* *6* *C–* *D*). Specifically, Wnt7a increased the proportion of Pax7^+^Ki67^+^ proliferative MuSCs at Day 3 (*Figure* *6* *C,D*). Correspondently, the proportion of emb‐MyHC^+^ newly formed myofibers increased in the Wnt7a group at Day 7 (*Figure* *S4*). In terms of fibrogenesis, Wnt7a delivery decreased the proportion of Pdgfra^+^ FAPs at Days 3, 7 and 14, resulting in a significantly reduced Picro Sirus Red‐positive fibrosis area (*Figure* *6* *F*).

## Discussion

Fibrosis frequently occurs after reconstructive lip surgeries, and surgical manoeuvres are believed to contribute to the formation of fibrous tissue.^8^ In this work, the effect of each single surgical manipulation on the healing process was dissected with the help of a rat OO muscle regeneration model. It provided direct evidence that tensile force proved to be the most detrimental factor.

In lip repair procedures, the application of tensile force is critical to achieving a desirable surgical outcome. Results from both clinical trials and finite element analysis revealed that appropriate force loading was essential in correcting cleft lip and nose deformities.^23^, ^24^ Otherwise, relapse would occur. Tensile force is of more importance in bilateral lip repair, where techniques to reconstruct the upper lip without restoring the continuity of the OO muscle to avoid excessive tension usually result in an excessively broad and vertically short philtrum.^3^, ^25^ Skin‐only closures will not provide the appropriate strength to narrow the cleft and may lead to significant landmark distortions and scarring,^6^ while overlapping sutures could generate greater inflammation and result in limited long‐term fullness.^26^ Moreover, force balance is required for post‐operative symmetry in surgical repair for bilateral cleft lips.^27^ In regard to the pros and cons of the tensile force in lip repair, a central question remains how cells compute a host of biomechanical signals into meaningful biological behaviours. If the key molecule regulating muscle regeneration in response to mechanical strains was identified, biotherapeutics targeting this molecule could be applied to counteract this pro‐fibrotic effect.

The influence of mechanical strains on cell behaviour has been investigated extensively. It has been reported that mechanical strain could promote adipose‐derived stem cell proliferation.^28^ In the context of wound healing, tensile force will drive a fibroblast‐to‐myofibroblast transition, together with increased cell proliferation and Yes‐associated protein (*Yap*) nuclear translocation.^29^ This would result in fibrosis and scar formation. However, in the process of skeletal muscle regeneration, it has been shown that mechanical strains exerted on C2C12 cells increased the level of reactive oxygen species and cell apoptosis, thus impairing the healing process.^30^ Our work demonstrated that stretched muscle had both increased fibrogenesis and inhibited myogenesis, in contrast to milder injuries such as dissection and transection. Surprisingly, the RNA‐seq analysis of MuSCs revealed that *Il10* played a central role in regulating OO muscle fibrosis. The major source of *Il10* was considered the anti‐inflammatory macrophages upon muscle injury.^31^ However, the expression levels of *Il10* in MuSCs and FAPs, two main stem cells responsible for skeletal muscle regeneration, were seldom investigated. The in vitro work from our study proved that both the *Il10* expression level and the IL‐10 protein concentration were significantly upregulated in MuSCs and FAPs after surgical injury. Blunting *Il10* expression with rh‐Wnt7a administration could ameliorate the injury‐induced OO muscle fibrosis.

The anti‐inflammatory cytokine Il10 has pleiotropic and even conflicting roles in mediating tissue fibrosis.^32^ Il10 can play either beneficial or maladaptive roles in fibrotic diseases in the skin, liver, intestine and pancreas, depending on the organ system, disease model or cell type examined.^31^, ^33^ In our surgery‐induced OO muscle regeneration models, the *Il10* expression level was always positively correlated with the fibrotic level. Similarly, in chronic limb muscle regeneration, *Il10* has been considered a pro‐fibrotic cytokine because Il10 could skew macrophages to an anti‐inflammatory type and provide substrate to fibroblasts for connective tissue production.^34^ Meanwhile, in the process of cardiac regeneration, Il10 is typically viewed as pro‐fibrotic. Hulsmans et al. reported that cardiac Il10 could activate fibroblasts, and deletion of *Il10* improves diastolic function.^33^ Taking into consideration the convergent roles of *Tgfb*, *Wnt* and *Yap/Taz* signalling in regulating tissue fibrosis, it is worthwhile to see how *Il10* regulates this stretch‐induced fibrosis in future studies.^35^

Another plausible explanation for the persistent OO muscle fibrosis might be the inferior regenerative capacities of the orofacial muscle. Previous experimental studies uncovered that mice masseter muscle had severely impaired muscle regeneration and prolonged fibrosis in comparison to tibialis anterior muscle, even when they were exposed to the same injury stimulus.^36^, ^37^ In the scenario of surgery‐induced injury, however, the comparison of orofacial muscle and limb muscle was not applicable. In fact, the orofacial muscles had unique anatomical relations with their surrounding structures: True myocutaneous junctions of skeletal muscle fibres are present only in the face.^38^ In cleft surgeries, wide undermining is necessary to facilitate a tension‐free skin reapproximating,^7^ which inevitably breaks off the myocutaneous junction. In contrast, in trunk and limb muscles, this junction does not exist and is thus not injured during surgeries. Therefore, it is more convincing to conduct studies in orofacial muscle models instead of trunk and limb muscles^26^ when exploring the effects of cleft‐related surgical techniques on muscle regeneration. And the relevant interventions to promote muscle regeneration and restrain fibrosis might be exclusive to the orofacial region.

Caution must be taken when interpretating the data acquired from the rat model due to the existing differences in both facial structures and muscle regeneration capacity between the two species. As we demonstrated in a previous study, although distinct anatomic and histologic features exist between rat and human lip epithelial tissue, the OO muscle of rats largely resembles that of humans.^16^ However, the regenerative capacity of skeletal muscle is much stronger in rats, resulting from the higher capacity of resorbing necrotic tissue and activating the inflammatory response in reaction to injuries.^39^ Therefore, the translatability of results from this rat model to human populations may be limited.

In conclusion, our study demonstrated the establishment and characterization of an animal model for surgical lip repair. Based on this experimental model, we figured out that stretching is the most detrimental factor to OO muscle regeneration. We also revealed that Il10 is a key regulator in OO muscle fibrosis, which could be targeted to ameliorate fibrotic tissue formation. This clinically oriented model will be useful for further investigations seeking adjunctive biotherapies to promote post‐operative orofacial muscle regeneration.

## Conflict of interest statement

Xu Cheng, Jinfeng Dou, Jinggui Li, Yixuan Huang, Bing Shi and Jingtao Li declare that they have no conflict of interest.

## Supporting information


**Table S1.** Experimental groups
**Table S2.** Parameters of mechanical strain application

**Figure S1.** Molecular comparison of muscle fibrosis and inflammation in different groups. Rat OO muscle was harvested at different timepoints after injury and quantitative real‐time PCR were performed to investigate the changes in marker genes of muscle fibrosis and tissue inflammation. (A)Quantification of relative expression level of *Tgfb1*. (B) Quantification of relative expression level of *Tnfa*. (C) Quantification of relative expression level of *Il6*. (D) Quantification of relative expression level of *Il1b*. *, *p* < 0.05; **, *p* < 0.01; ***, *p* < 0.001.

**Figure S2.** Fluorescence activated cell sorting of rat OO MuSCs. Cells were isolated at 4 days after surgical injury. Flow cytometry was performed to sort the Vcam1^+^Sca1^−^ MuSCs cell clusters. (A) Gating strategy of the Vcam1^+^Sca1^−^ cells in blank, dissection, transection and stretch group. (B) Pax7 immunofluorescence staining confirmed the high purity of sorted MuSCs. Scale bar, 100um.

**Figure S3.** *Il10* expression was upregulated in FAPs from injured groups. (A) Pdgfra immunofluorescent staining confirmed high purity of pre‐plated FAPs. (B) Relative expression level of *Il10* and IL‐10 protein concentration in FAPs from different groups. Scale bar, 100um.

**Figure S4.** Assessment of myogenesis in stretched muscle after rh‐Wnt7a administration. (A)Immunofluorescent staining of DAPI (blue), emb‐MyHC (red) and laminin (green) in different groups. (B) Quantification of emb‐MyHC^+^ myofibers. Scale bar, 200um. *, *p* < 0.05; ***, *p* < 0.001.

## References

[1] Park JA , Rho NK , Lee HI , Yeo IS , Koh KS , Song WC . Are there other muscle fibers on the orbicularis oris muscle in the upper lip? Plast Reconstr Surg 2022;150:1314e–1321e. 10.1097/PRS.0000000000009685 36161795

[2] Noor RAM , Shah NSM , Zin AAM , Sulaiman WAW , Halim AS . Disoriented collagen fibers and disorganized, fibrotic orbicularis oris muscle fiber with mitochondrial myopathy in non‐syndromic cleft lip. Arch Oral Biol 2022;140:105448. 35550192 10.1016/j.archoralbio.2022.105448

[3] Martin SV , Van Eeden S , Swan MC . Secondary surgery techniques to optimise functional and aesthetic outcomes in orofacial clefting. Br Dent J 2023;234:899–905. 37349438 10.1038/s41415-023-6001-8

[4] Denadai R , Chou PY , Pascasio DCG , Lo LJ . Modified unilateral incomplete cleft lip repair with primary nasal overcorrection: a muscle‐driven technique. Plast Reconstr Surg 2021;147:700–705. 33620940 10.1097/PRS.0000000000007688

[5] Zhang C , Yao M , Low DW , Wu M , Shi B , Zheng Q , et al. Outcome comparisons of two different orbicularis oris muscle reconstruction techniques in patients with unilateral incomplete cleft lip. Plast Reconstr Surg 2023. 10.1097/PRS.0000000000010855 37337337

[6] Xue AS , Buchanan EP , Hollier LH . Update in unilateral cleft lip surgery. Plast Reconstr Surg 2021;148:262e–274e. 34398098 10.1097/PRS.0000000000008141

[7] Xue AS , Buchanan EP , Hollier LH Jr . Bilateral cleft lip repair: lessons from history. Plast Reconstr Surg 2022;150:201e–210e. 10.1097/PRS.0000000000009241 35767636

[8] Szyszka‐Sommerfeld L , Machoy ME , Wilczynski S , Lipski M , Wozniak K . Superior orbicularis oris muscle activity in children surgically treated for bilateral complete cleft lip and palate. J Clin Med. 2021;10. 10.3390/jcm10081720 PMC8074006 33923491

[9] Wosczyna MN , Rando TA . A muscle stem cell support group: coordinated cellular responses in muscle regeneration. Dev Cell 2018;46:135–143. 30016618 10.1016/j.devcel.2018.06.018 PMC6075730

[10] Cheng X , Huang Y , Liu Y , Dou J , Zhao N , Li J , et al. Head muscle fibro‐adipogenic progenitors account for the tilted regeneration towards fibrosis. Biochem Biophys Res Commun 2022;589:131–138. 34915407 10.1016/j.bbrc.2021.12.009

[11] Vyas B , Nandkishore N , Sambasivan R . Vertebrate cranial mesoderm: developmental trajectory and evolutionary origin. Cell Mol Life Sci 2020;77:1933–1945. 31722070 10.1007/s00018-019-03373-1 PMC11105048

[12] Schubert FR , Singh AJ , Afoyalan O , Kioussi C , Dietrich S . To roll the eyes and snap a bite—function, development and evolution of craniofacial muscles. Semin Cell Dev Biol 2019;91:31–44. 29331210 10.1016/j.semcdb.2017.12.013

[13] Rosero Salazar DH , Carvajal Monroy PL , Wagener F , Von den Hoff JW . Orofacial muscles: embryonic development and regeneration after injury. J Dent Res 2020;99:125–132. 31675262 10.1177/0022034519883673 PMC6977159

[14] Diogo R , Kelly RG , Christiaen L , Levine M , Ziermann JM , Molnar JL , et al. A new heart for a new head in vertebrate cardiopharyngeal evolution. Nature 2015;520:466–473. 25903628 10.1038/nature14435 PMC4851342

[15] Hardy D , Besnard A , Latil M , Jouvion G , Briand D , Thépenier C , et al. Comparative study of injury models for studying muscle regeneration in mice. PLoS ONE 2016;11:e0147198. 26807982 10.1371/journal.pone.0147198 PMC4726569

[16] Li J , Huang Y , Li J , Shi B , Cheng X . A novel rat model for muscle regeneration and fibrosis studies in surgical lip repair. Cleft Palate‐Craniofa J 2022;10556656221136171. 10.1177/10556656221136171 36341784

[17] Meng H , Janssen PM , Grange RW , Yang L , Beggs AH , Swanson LC , et al. Tissue triage and freezing for models of skeletal muscle disease. J Vis Exp 2014;89. 10.3791/51586 PMC4215994 25078247

[18] Carvajal Monroy PL , Yablonka‐Reuveni Z , Grefte S , Kuijpers‐Jagtman AM , Wagener FA , Von den Hoff JW . Isolation and characterization of satellite cells from rat head branchiomeric muscles. J Vis Exp 2015;e52802. 26274878 10.3791/52802 PMC4544364

[19] Liu L , Cheung TH , Charville GW , Rando TA . Isolation of skeletal muscle stem cells by fluorescence‐activated cell sorting. Nat Protoc 2015;10:1612–1624. 26401916 10.1038/nprot.2015.110 PMC4793971

[20] Charan J , Kantharia ND . How to calculate sample size in animal studies? J Pharmacol Pharmacother 2013;4:303–306. 24250214 10.4103/0976-500X.119726 PMC3826013

[21] Chae WJ , Bothwell ALM . Canonical and non‐canonical wnt signaling in immune cells. Trends Immunol 2018;39:830–847. 30213499 10.1016/j.it.2018.08.006 PMC7367500

[22] Cheng X , Huang H , Luo X , Shi B , Li J . Wnt7a induces satellite cell expansion, myofiber hyperplasia and hypertrophy in rat craniofacial muscle. Sci Rep 2018;8:10613. 30006540 10.1038/s41598-018-28917-6 PMC6045621

[23] Shi B , Huang H . Computational technology for nasal cartilage‐related clinical research and application. Int J Oral Sci 2020;12:21. 32719336 10.1038/s41368-020-00089-y PMC7385163

[24] Huang H , Li Y , Luo X , Cheng X , Shi B , Li J . Mechanical analyses of critical surgical maneuvers in the correction of cleft lip nasal deformity. PLoS ONE 2018;13:e0195583. 29652906 10.1371/journal.pone.0195583 PMC5898757

[25] Manchester WM . The repair of bilateral cleft lip and palate. Br J Surg 1965;52:878–882. 5842977 10.1002/bjs.1800521111

[26] Kim J , Choi J , Kim J , Jo T , Hwang I , Han K , et al. An evaluation of muscle repair techniques: implications in musculoskeletal healing and corollaries in oral‐facial clefting. J Clin Med 2021;10:4803. 34768323 10.3390/jcm10214803 PMC8584801

[27] Huang H , Han Y , Akinade T , Li J , Shi B , Li C . Force balance reconstruction of the orbicularis oris in unilateral incomplete cleft lip. J Plast Reconstr Aesthet Surg 2020;73:1717–1722. 32446569 10.1016/j.bjps.2020.03.010

[28] Chen X , Deng Z , He Y , Lu F , Yuan Y . Mechanical strain promotes proliferation of adipose‐derived stem cells through the integrin β1‐mediated RhoA/myosin light chain pathway. Tissue Eng Part A 2020;26:939–952. 32066340 10.1089/ten.TEA.2019.0266

[29] Kollmannsberger P , Bidan CM , Dunlop JWC , Fratzl P , Vogel V . Tensile forces drive a reversible fibroblast‐to‐myofibroblast transition during tissue growth in engineered clefts. Sci Adv 2018;4:eaao4881. 29349300 10.1126/sciadv.aao4881 PMC5771696

[30] Yi Y , Wang L , Li S , Li B , Liu C , Hong L . Effects of mechanical trauma on the differentiation and ArfGAP3 expression of C2C12 myoblast and mouse levator ani muscle. Int Urogynecol J 2020;31:1913–1924. 31989201 10.1007/s00192-019-04212-4

[31] Tidball JG . Regulation of muscle growth and regeneration by the immune system. Nat Rev Immunol 2017;17:165–178. 28163303 10.1038/nri.2016.150 PMC5452982

[32] Steen EH , Wang X , Balaji S , Butte MJ , Bollyky PL , Keswani SG . The role of the anti‐inflammatory cytokine interleukin‐10 in tissue fibrosis. Adv Wound Care (New Rochelle) 2020;9:184–198. 32117582 10.1089/wound.2019.1032 PMC7047112

[33] Hulsmans M , Sager HB , Roh JD , Valero‐Muñoz M , Houstis NE , Iwamoto Y , et al. Cardiac macrophages promote diastolic dysfunction. J Exp Med 2018;215:423–440. 29339450 10.1084/jem.20171274 PMC5789416

[34] Tidball JG , Welc SS , Wehling‐Henricks M . Immunobiology of inherited muscular dystrophies. Compr Physiol 2018;8:1313–1356. 30215857 10.1002/cphy.c170052 PMC7769418

[35] Piersma B , Bank RA , Boersema M . Signaling in fibrosis: TGF‐β, WNT, and YAP/TAZ converge. Front Med (Lausanne) 2015;2:59. 26389119 10.3389/fmed.2015.00059 PMC4558529

[36] Yoshioka K , Kitajima Y , Seko D , Tsuchiya Y , Ono Y . The body region specificity in murine models of muscle regeneration and atrophy. Acta Physiol (Oxf) 2021;231:e13553. 32875719 10.1111/apha.13553 PMC7757168

[37] Pavlath GK , Thaloor D , Rando TA , Cheong M , English AW , Zheng B . Heterogeneity among muscle precursor cells in adult skeletal muscles with differing regenerative capacities. Dev Dyn 1998;212:495–508. 9707323 10.1002/(SICI)1097-0177(199808)212:4<495::AID-AJA3>3.0.CO;2-C

[38] May CA , Bramke S . In the human, true myocutaneous junctions of skeletal muscle fibers are limited to the face. J Anat 2021;239:445–450. 33641167 10.1111/joa.13419 PMC8273604

[39] Borisov AB . Regeneration of skeletal and cardiac muscle in mammals: do nonprimate models resemble huamn pathology? Wound Repair Regen 1999;7:26–35. 10231503 10.1046/j.1524-475x.1999.00026.x

[40] von Haehling S , Morley JE , Coats AJS , Anker SD . Ethical guidelines for publishing in the *Journal of Cachexia, Sarcopenia and Muscle*: update 2021. J Cachexia Sarcopenia Muscle 2021;12:2259–2261. 34904399 10.1002/jcsm.12899 PMC8718061

