# Supplementary material for: Tensile force impairs lip muscle regeneration under the regulation of interleukin‐10
Source: J Cachexia Sarcopenia Muscle. 2024 Oct 1;15(6):2497–508. doi: 10.1002/jcsm.13584 (PMC11634486; doi:10.1002/jcsm.13584)
Supplement: Supplementary file 1 — Table S1. Experimental groups Table S2. Parameters of mechanical strain application [file JCSM-15-2497-s004.docx]

**Tensile force impairs lip muscle regeneration under the regulation of interleukin-10**

Xu Cheng^1,#^ , **Jinfeng Dou^2, #^** , **Jinggui Li^1,3^**, Yixuan Huang^2^, **Bing Shi^2,*^ , Jingtao Li^2,*^**

^1^State Key Laboratory of Oral Diseases & National Center for Stomatology & National Clinical Research Center for Oral Diseases, West China Hospital of Stomatology, Sichuan University, Chengdu 610041, Sichuan, China

^2^State Key Laboratory of Oral Diseases & National Center for Stomatology & National Clinical Research Center for Oral Diseases & Department of Oral and Maxillofacial Surgery, West China Hospital of Stomatology, Sichuan University, Chengdu 610041, Sichuan, China

^3^Department of Oral and Craniomaxillofacial Surgery, Shanghai Ninth People’s Hospital, Shanghai Jiao Tong University School of Medicine; College of Stomatology, Shanghai Jiao Tong University; National Center for Stomatology; National Clinical Research Center for Oral Diseases; Shanghai Key Laboratory of Stomatology, Shanghai,200011, China

Xu Cheng [chengxk10727002@163.com](mailto:chengxk10727002@163.com) Jinfeng Dou [doujinfengkq@163.com](mailto:doujinfengkq@163.com)

Jinggui Li [jingguiliqqzj@163.com](mailto:jingguiliqqzj@163.com) Yixuan Huang hyxnoemie96@126.com

^#^Authors contributing equally to this article.

Corresponding authors:

Bing Shi [shibingcn@vip.sina.com](mailto:shibingcn@vip.sina.com)

State Key Laboratory of Oral Diseases, National Clinical Research Center for Oral Diseases, Department of Oral & Maxillofacial Surgery, West China Hospital of Stomatology, Sichuan University, No. 14, Section 3, South Renmin Rd., Wuhou District, Chengdu 610041, China.

Jingtao Li [lijingtao86@163.com](mailto:lijingtao86@163.com)

State Key Laboratory of Oral Diseases, National Clinical Research Center for Oral Diseases, Department of Oral & Maxillofacial Surgery, West China Hospital of Stomatology, Sichuan University, No. 14, Section 3, South Renmin Rd., Wuhou District, Chengdu 610041, China.

**Appendix Table S1. Experimental groups**

| **Surgical status** | **Harvest time** | **Sample size** | **Analysis** | **Figure(s)** |
| --- | --- | --- | --- | --- |
| Blank | 3 dpi | 6 | Histology and immunofluorescent staining, QPCR | 1,2,3,6 |
|  | 7 dpi | 6 |  |  |
|  | 14 dpi | 6 |  |  |
|  | 21 dpi | 6 |  |  |
|  | 56 dpi | 3 |  |  |
| Dissection | 3 dpi | 6 | Histology and immunofluorescent staining, QPCR | 1,2,3 |
|  | 7 dpi | 6 |  |  |
|  | 14 dpi | 6 |  |  |
|  | 21 dpi | 6 |  |  |
|  | 56 dpi | 3 |  |  |
| Transection | 3 dpi | 6 | Histology and immunofluorescent staining, QPCR |  |
|  | 7 dpi | 6 |  |  |
|  | 14 dpi | 6 |  |  |
|  | 21 dpi | 6 |  |  |
|  | 56 dpi | 3 |  |  |
| Stretch | 3 dpi | 6 | Histology and immunofluorescent staining, QPCR | 1,2,3,6 |
|  | 7 dpi | 6 |  |  |
|  | 14 dpi | 6 |  |  |
|  | 21 dpi | 6 |  |  |
|  | 56 dpi | 3 |  |  |
| Stretch + rh-Wnt7a | 3 dpi | **9** | Histology and immunofluorescent staining, **ELISA** | 6 |
|  | 7 dpi | 6 |  |  |
|  | 14 dpi | 6 |  |  |
|  | 21 dpi | 6 |  |  |
|  | 56 dpi | 3 |  |  |
| Blank | 4 dpi | **9** | RNA-seq, cell culture, QPCR,  **ELISA** | 5 |
| Dissection | 4 dpi | **9** |  |  |
| Transection | 4 dpi | **9** |  |  |
| Stretch | 4 dpi | **9** |  |  |
| Blank | 0 dpi | 9 | Cell proliferation and differentiation after mechanical strain application | 4 |

**Appendix Table S2. Parameters of mechanical strain application**

|  | Proliferation | | | | Differentiation | | | |
| --- | --- | --- | --- | --- | --- | --- | --- | --- |
| d(mm) | 0.0 | 0.5 | 1.0 | 2.0 | 0.0 | 0.5 | 1.0 | 2.0 |
| ε(µ strain) | 0 | 672 | 1344 | 2688 | 0 | 672 | 1344 | 2688 |
| Frequency (Hz) | 0.5 | 0.5 | 0.5 | 0.5 | 0.5 | 0.5 | 0.5 | 0.5 |
| Duration (min) | 120 | 120 | 120 | 120 | 120 | 120 | 120 | 120 |
| Rest periods (h) | 22 | 22 | 22 | 22 | 22 | 22 | 22 | 22 |
| Stretching days (d) | 1 | 1 | 1 | 1 | 3 | 3 | 3 | 3 |

**Appendix Figure S1. Molecular comparison of muscle fibrosis and inflammation in different groups.** Rat OO muscle was harvested at different timepoints after injury and quantitative real-time PCR were performed to investigate the changes in marker genes of muscle fibrosis and tissue inflammation. (A)Quantification of relative expression level of *Tgfb1*. (B) Quantification of relative expression level of *Tnfa*. (C) Quantification of relative expression level of *Il6*. (D) Quantification of relative expression level of *Il1b*. ***, *p*<0.05; **, *p*<0.01; ***, *p*<0.001.**

**Appendix Figure S2. Fluorescence activated cell sorting of rat OO MuSCs.** Cells were isolated at 4 days after surgical injury. Flow cytometry was performed to sort the Vcam1^+^Sca1^-^ MuSCs cell clusters. (A) Gating strategy of the Vcam1^+^Sca1^-^ cells in blank, dissection, transection and stretch group. (B) Pax7 immunofluorescence staining confirmed the high purity of sorted MuSCs. Scale bar, 100um.

**Appendix Figure S3. *Il10* expression was upregulated in FAPs from injured groups.** (A) Pdgfra immunofluorescent staining confirmed high purity of pre-plated FAPs. (B) Relative expression level of *Il10* **and IL-10 protein concentration** in FAPs from different groups. Scale bar, 100um.

**Appendix Figure S4. Assessment of myogenesis in stretched muscle after rh-Wnt7a administration.** (A)Immunofluorescent staining of DAPI(blue), emb-MyHC(red) and laminin(green) in different groups. (B) Quantification of emb-MyHC^+^ myofibers. Scale bar, 200um. ***, *p*<0.05; ***, *p*<0.001.**
