# Supplementary figures and images for: Tensile force impairs lip muscle regeneration under the regulation of interleukin‐10
Source: J Cachexia Sarcopenia Muscle. 2024 Oct 1;15(6):2497–508. doi: 10.1002/jcsm.13584 (PMC11634486; doi:10.1002/jcsm.13584)

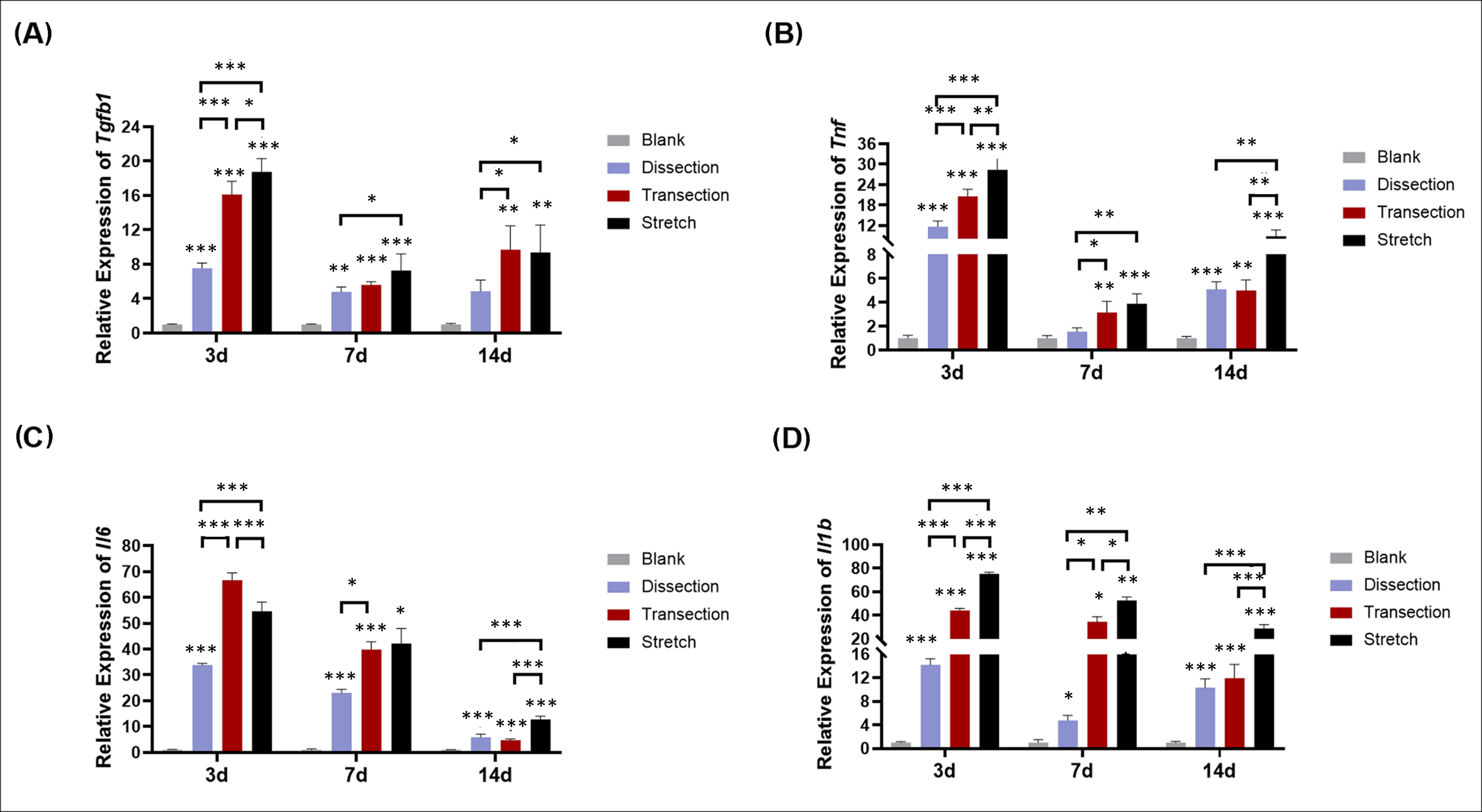

Supplement: Supplementary file 2 — Figure S1. Molecular comparison of muscle fibrosis and inflammation in different groups. Rat OO muscle was harvested at different timepoints after injury and quantitative real‐time PCR were performed to investigate the changes in marker genes of muscle fibrosis and tissue inflammation. (A)Quantification of relative expression level of Tgfb1. (B) Quantification of relative expression level of Tnfa. (C) Quantification of relative expression level of Il6. (D) Quantification of relative expression level of Il1b. *, p < 0.05; **, p < 0.01; ***, p < 0.001. [file JCSM-15-2497-s001.tif]

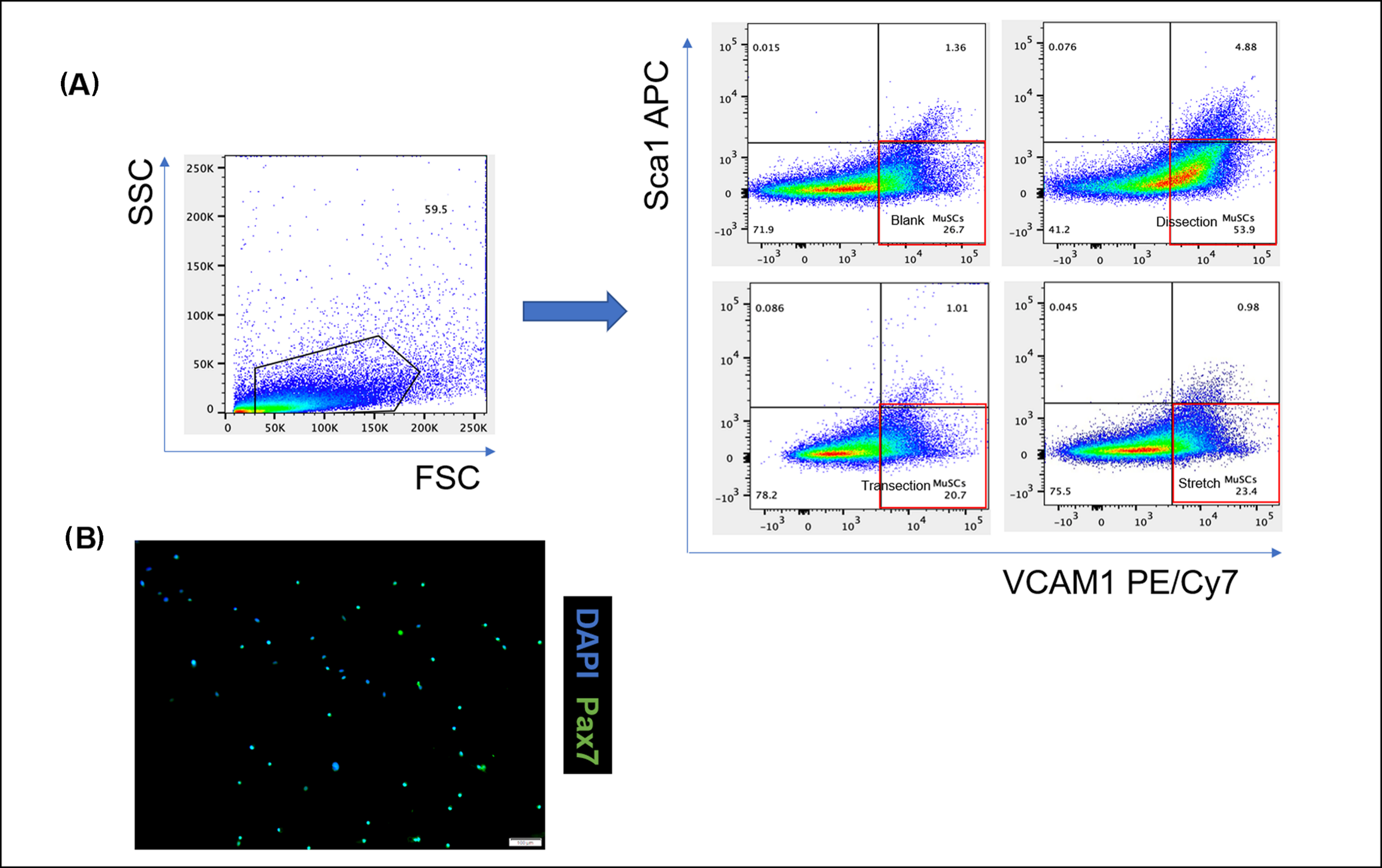

Supplement: Supplementary file 3 — Figure S2. Fluorescence activated cell sorting of rat OO MuSCs. Cells were isolated at 4 days after surgical injury. Flow cytometry was performed to sort the Vcam1+Sca1− MuSCs cell clusters. (A) Gating strategy of the Vcam1+Sca1− cells in blank, dissection, transection and stretch group. (B) Pax7 immunofluorescence staining confirmed the high purity of sorted MuSCs. Scale bar, 100um. [file JCSM-15-2497-s002.tif]

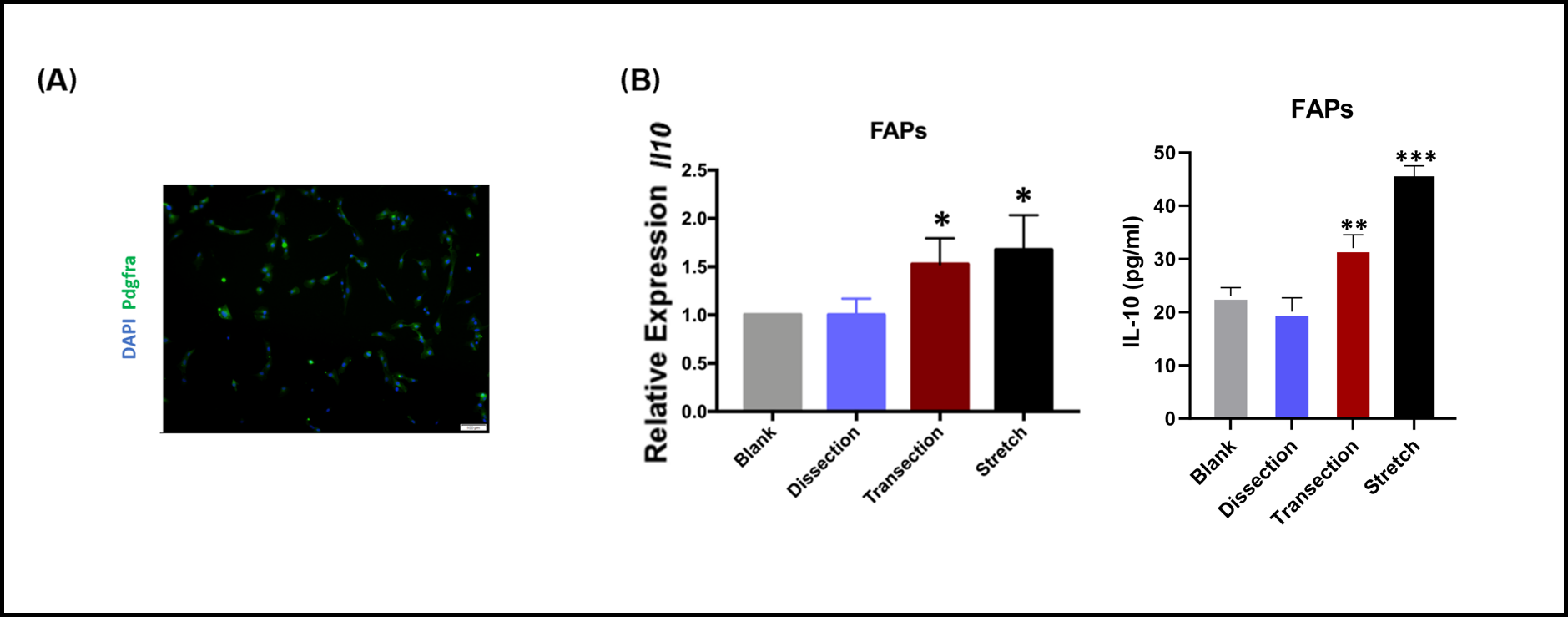

Supplement: Supplementary file 4 — Figure S3. Il10 expression was upregulated in FAPs from injured groups. (A) Pdgfra immunofluorescent staining confirmed high purity of pre‐plated FAPs. (B) Relative expression level of Il10 and IL‐10 protein concentration in FAPs from different groups. Scale bar, 100um. [file JCSM-15-2497-s003.tif]

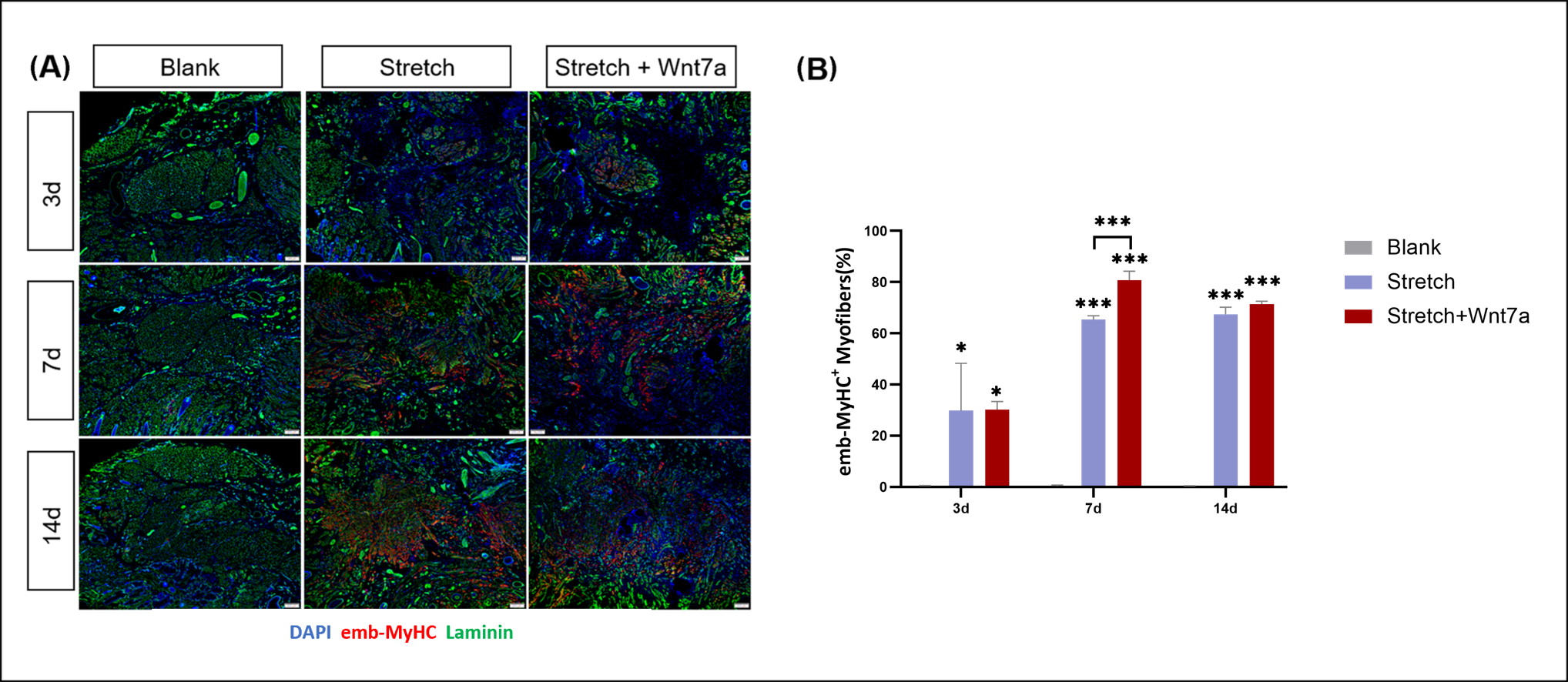

Supplement: Supplementary file 5 — Figure S4. Assessment of myogenesis in stretched muscle after rh‐Wnt7a administration. (A)Immunofluorescent staining of DAPI (blue), emb‐MyHC (red) and laminin (green) in different groups. (B) Quantification of emb‐MyHC+ myofibers. Scale bar, 200um. *, p < 0.05; ***, p < 0.001. [file JCSM-15-2497-s005.tif]
